# Phytosomal delivery enhances bioactivity of *Hylocereus costaricensis* phenolic extract

**DOI:** 10.3389/fnut.2025.1659572

**Published:** 2025-08-14

**Authors:** Rosa Direito, Inês Sousa, Filipa Antunes, Sandra Maria Barbalho, Sandra Simões, Maria Rosário Bronze, Catarina Reis, Maria Manuela Gaspar, Maria Eduardo Figueira

**Affiliations:** ^1^Laboratory of Systems Integration Pharmacology, Clinical and Regulatory Science, Research Institute for Medicines (iMed.ULisboa), Universidade de Lisboa, Lisbon, Portugal; ^2^Faculty of Pharmacy, Universidade de Lisboa, Lisbon, Portugal; ^3^Department of Biochemistry and Pharmacology, School of Medicine, University of Marília (UNIMAR), São Paulo, Brazil; ^4^Postgraduate Program in Structural and Functional Interactions in Rehabilitation, University of Marília (UNIMAR), São Paulo, Brazil; ^5^Department of Biochemistry and Nutrition, School of Food and Technology of Marília (FATEC), Saõ Paulo, Brazil; ^6^Charity Hospital, UNIMAR (HBU), University of Marília (UNIMAR), São Paulo, Brazil; ^7^ITQB, Oeiras, Portugal; ^8^IBET, Oeiras, Portugal

**Keywords:** phytosomes, phenolic extract, bioavailability, antioxidant, antiinflamatory activity

## Abstract

**Introduction:**

Consuming foods rich in bioactive compounds can delay chronic non-communicable diseases. Dragon fruit, known for its bioactive compounds with typically low bioavailability—attributed to high polarity, poor permeability, and rapid firstpass metabolism—benefits from strategies like encapsulation to enhance efficacy.

**Methods:**

This study developed and assessed phytosomes loaded with dragon fruit extract (Hylocereus costaricensis) and compared their biological activity *in vitro* and *in vivo* against the raw extract. Spectrophotometric analysis of the extract revealed 410.4 mg GAE/L of total phenolic compounds and 139.3 mg CE/L of flavonoids. HPLC-DAD-MS/MS identified key components such as citric acid, succinic acid, cyanidin-3-O-rutinoside, ferulic acid, betanin, and rutin. Phytosomes encapsulated 46 ± 2% of phenolics, with a mean diameter of 1,329 ± 121.0 nm, PDI 0.633 ± 0.039, and *ζ*-potential −16 ± 1 mV.

**Results:**

Antioxidant capacities, assessed by the DPPH method, showed that the phytosomal formulation with 46% phenolic content was as effective as the raw extract. *In vivo* antihyperglycemic studies showed no significant effect from the raw extract (5 mg GAE/kg), but phytosomes (2.3 mg GAE/kg) matched the efficacy of metformin (300 mg/kg). An acute inflammation model using carrageenan-induced paw edema indicated that phytosomes (2.3 mg GAE/kg) had superior anti-inflammatory effects compared to the raw extract (5 mg GAE/kg).

**Discussion:**

In summary, dragon fruit extract has antioxidant, antihyperglycemic, and anti-inflammatory properties. These findings support that phytosomal encapsulation significantly improves the bioavailability and efficacy of phenolic-rich crude extract, highlighting their potential in functional food development, nutraceutical applications, and as helpers to conventional therapeutic strategies.

## 1 Introduction

The (*Hylocereus* sp.), also known as dragon fruit, is a hardy fruit from the Cactaceae family under the genus *Hylocereus*, and this fruit is also referred to by various names, including, dragon pearl fruit, night-blooming cereus, strawberry pear, and Cinderella plant. The characteristics of its fruits, such as shape, thorn presence, and the color of the skin and pulp, vary among species, demonstrating significant genetic diversity (1–3).

The most cultivated species are: *Hylocereus undatus*, with a red shell and white flesh; *Hylocereus polyrhizus*, with a red skin and red flesh; *Hylocereus costaricensis*, with a red rind and red-violet flesh; *Selenicereus megalanthus* (Syn. *Hylocereus megalanthus*), with a yellow rind with spines and white flesh (3, 4).

The phytochemical compounds found in dragon fruit mainly belong to phenols, gallic acid, ferulic acid, quercetin, rutin, and betanin. Among the numerous bioactive compounds found in the pulp and peel, we can highlight the presence of ascorbic acid, tocopherol, thiamin, niacin, riboflavin, betacyanin, *β*-carotene, lycopene, p-coumaric acid, protocatechuic acid, vanillic acid, gallic acid, syringic acid, p-hydroxybenzoic acid, and minerals like calcium and phosphorous (3, 5–8).

(*Hylocereus* sp.) extracts have been shown to have various properties: antioxidant, anti-diabetic, antiviral and antimicrobial, anti-cancer, healing, antihyperlipidemic and anti-obesity, hepatoprotective, anti-inflammatory, anti-anemic and prebiotic (9). The is considered a natural agent that prevents diseases associated with ageing, mainly related to oxidative stress due to the imbalance between antioxidants and free radicals, such as cancer, diabetes, hypertension, Alzheimer’s disease, and Parkinson’s disease (9–15).

It has been the subject of scientific studies into its anti-diabetic properties and its ability to regulate glycemic levels (antihyperglycemic) (16–21). Many studies have demonstrated that the use of red can reduce glucose blood levels in humans (21–23). Ajie (17) have mentioned flavonoid content mediating hypoglycemic action via 3 mechanisms, namely, decreasing oxidative stress by antioxidant effect, inhibition of intestinal mucosal GLUT 2 and inhibition of phosphodiesterase, thereby increasing insulin retention in patients. Marietta et al. (24) studied the effects of red pitaya skin extract on the glycemia and lipid profiles of diabetic and dyslipidemic male Wistar rats and found no significant reduction in glycemia. In a systematic review and meta-analysis, Poosulp et al. (25) found that dragon fruit can be used to prevent diabetes; however, Poolsup and colleagues concluded that the effect in T2DM patients was not significant. Then again, a trend towards greater blood glucose reduction with higher dose was observed (25). However, higher doses have risk factors regarding the antioxidant effect, can become pro-oxidant effects, and toxicity doses could be reached. With the use of drug delivery systems for pitahaya’s compounds encapsulation, the release of bioactive compounds in efficient doses could be achieved in a perfectly controlled way. The efficiency of digestion, absorption and metabolization of phenolic compounds depends on their bioaccessibility and bioavailability. Thus, the greater the bioaccessibility and bioavailability of a phenolic compound in the body, the greater the health benefits (26, 27). Nevertheless, none of these studies applied drug delivery systems for pitahaya’s compounds. Encapsulation of extracts containing phenolic compounds can be a solution to the low bioaccessibility and bioavailability of these compounds, as it makes it possible to achieve the defined plasma concentrations needed to trigger their biological effect in the body (28, 29), while at the same time preserving the integrity of these compounds in environments with adverse conditions along the gastrointestinal tract.

Betacyanins in the *Hylocereus* species are natural water-soluble nitrogenous pigments with red-violet color. Together with yellow betaxanthins, they constitute betalains, an abundant group of ‘s phytochemicals. However, as with other bioactive compounds, betacyanins exhibit a short shelf-life and low bioavailability (some studies show that betalains bioavailability is lower than 1% of the ingested amount). Betalains bioavailability was found to be around 0.5–0.9% among human volunteers (30). Sawick et al. (31) revealed that betacyanins suffered intensive degradation when fermented juice prepared with red beet was administered by oral gavage to rats and absorbed from the stomach. Nineteen betacyanins (eight were native compounds, and 11 were metabolites) were found in the physiological fluids of the rats. Krant et al. (32) showed that betanin underwent metabolization mainly in the stomach wall (74%), followed by the colon (60%), and in a small percentage, in the small intestine (35%).

Furthermore, the presence of these compounds is influenced by external factors, such as food source, food matrix (type of product, structure, composition, processing, and viscosity), processing (pH, viscosity, and fermentation), water activity, temperature, oxygen, light, mechanisms of absorption, and catalytic enzymes (30, 33–39). The preparation of nanoliposome phospholipids augmented the stability of betanin and resulted in higher 2,2-diphenyl-1-picrylhydrazyl radical-scavenging capacity in gummy candies than those containing free betanin (36). Encapsulated betalain showed higher encapsulation efficiency and emulsion stability (38). In the hydrophilic form, betacyanin from red dragon fruit extract is a high-value bioactive component with a plethora of applications as functional food or nutraceuticals. Harimurti et al. (34) developed a red dragon fruit extract encapsulated product in water-in-oil-in-water nanoemulsion as a delivery system ensuring antioxidant activities. In another study, the betalains from pitahaya microencapsulation added of potato succinylated starch increased the stability of stored yogurt (40). Rizvi et al. (41) worked on the green synthesis of iron oxide nanoparticles made from fruit extract of this species and observed efficient photocatalytic actions towards azo dyes. Alginate microspheres loaded with betacyanins from the bark of *Hylocereus polyrhizus* were effective as additives, food colorings and for oral administration (29).

Al-Radadi et al. (15) reported that gold nanoparticles synthesized with *H. polyrhizus* pulp and seed oil extract exhibited notable anti-inflammatory, anti-diabetic, anti-Alzheimer, and cytotoxic properties. *In vitro*, the nanoparticles (50–200 mg/mL) significantly inhibited alpha-amylase, demonstrating anti-diabetic effects; maximum anti-inflammatory activity was observed at 400 mg/mL, and antioxidant activity increased with higher concentrations (15). These results bring to light the possibility of using the genus *Hylocereus* to produce nanoparticles that can be used for pharmaceutical purposes or in the food industry. Producing well-researched and controlled food supplements by encapsulating extracts, rich in betalains, flavonoids, vitamin C, and other phytochemicals, addresses the challenges of low bioavailability and effective dosing of bioactive compounds, thereby enhancing consumer health. This approach also extends the shelf life of these valuable compounds, derived from a perishable, seasonal, and often expensive fruit that is rarely available in national markets. When it is available, it is typically too costly for the general population. Encapsulation makes these health benefits more accessible and affordable.

However, the field of nanotechnology applying extracts is still embryonic (although promising and showing great potential), and several technologies still need to be tested.

This study aims to develop and evaluate phytosomes with dragon fruit extract (*Hylocereus costaricensis*), assess their *in vitro* biological effects, and compare their *in vivo* activity to the free extract. Results indicate that the phenolic extract possesses antioxidant, anti-hyperglycemic, and anti-inflammatory properties, suggesting potential health benefits when included in the diet, with phytosomes enhancing these effects.

## 2 Materials and methods

### 2.1 Materials, solvents and reagents

Absolute ethanol (99.5%) from Fisher Scientific (Loughborough, UK); distilled water from Desminágua (Cascais, Portugal) and compresses from Hassemed (Porto, Portugal); Folin–Ciocalteu reagent from Merck KGaA (Darmstadt, Germany); sodium carbonate (99.9%) from VWR (Leuven, Belgium); gallic acid (97.5%) from Sigma-Aldrich (St. Louis, MO, US); quartz cells with an optical path of 1 cm; catechin (98%) from Sigma-Aldrich (St. Louis, MO, USA); sodium nitrite (98%) from Panreac Quimica (Barcelona, Spain); aluminum chloride (99%) from Chem-Lab NV (Zedelgem, Belgium); sodium hydroxide (1 M) from Fisher Chemical (Loughborough, UK); methanol (99.9%) from Honeywell Riedelde-Haën (Seelze, Germany); metaphosphoric acid (39–43%) from Thermo Scientific (Waltham, MA, USA); sodium acetate trihydrate (99–101%) from Panreac Quimica (Barcelona, Spain); glacial acetic acid (99.7%) from Fisher Scientific (Loughborough, UK); 2,6-diclophenol indophenol sodium salt (90%) from Riedel-de Haën (Charlotte, NC, USA); sodium hydrogen carbonate (99.7–100.3%) from Fisher Scientific (Loughborough, UK); ascorbic acid (99.7%) from Panreac Quimica (Barcelona, Spain); xylene from Fisher Scientific (Loughborough, UK); filter paper (Whatman, n°1); formic acid from Fluka (Seelze, Germany); acetonitrile HPLC gradient grade from VWR (Leuven, Belgium); MilliQ Millipore water from Direct Q3 UV System equipment (Molsheim, France). Human keratinocyte cell line HaCaT from Cell line Service GmbH (Eppelheim, Germany); standard phosphate buffer (PBS) from Sigma-Aldrich (St. Louis, MO, USA); 2′,7′-dichlorodihydrofluorescein diacetate (H2-DCFDA) from Life Technologies (Warrington, UK); ascorbic acid (99.7%) from Panreac Quimica (Barcelona, Spain); hydrogen peroxide (H_2_O_2_) from Merck (Darmstadt, Germany).

Phosphatidylcholine (60% purified from the yolk of egg) from Sigma-Aldrich (St. Louis, MO, USA); glacial acetic acid (99.7%) from Fisher Scientific (Loughborough, UK); absolute ethanol (99.5%) from Fisher Scientific (Loughborough, UK). Sunflower oil from Riazor (Santarém, Portugal); 2.2-diphenyl-1-picrylhydrazyl (DPPH) from Sigma-Aldrich (St. Louis, MO, USA); quercetin (95%) from Sigma-Aldrich (St. Louis, MO, USA).

Glucose from Sigma-Aldrich (St. Louis, MO, USA); metformin from Generis Farmacêutica S. A. (Amadora, Portugal); Codan brand gavage syringes (Odivelas, Portugal); ketamine (Imalgene 1,000) and xylazine (Rompun 2%) from Bio2 Veterinary Products (Lisbon, Portugal). Medetomidine from CP-Pharma (Handelsges, Germany); *λ* - carrageenan from Sigma-Aldrich (St. Louis, MO, USA); sterile saline solution from B. Braun Portugal (Queluz de Baixo, Portugal); isoflurane isovet from B. Braun Portugal (Queluz de Baixo, Portugal).

### 2.2 Equipments

Scales from Kern PCB (Balingen, Germany) and Mettler Toledo (Columbus, OH, USA); blender Ft1725 from Electric Co. (Lisbon, Portugal); vibro-fix vortex from Ika (Staufen, Germany); centrifuges Janetzki T 32 (Wallhausen, Germany) and Heraeus Biofuge Stratos from Kendro Laboratory Products (Osterode, Germany); rotavapor Laborota 4,001 from Heidolph (Schwabach, Germany); Hitachi U-2000 spectrophotometer (Tokyo, Japan); heating chamber DOVF-125-001 from Labbox (Barcelona, Spain); liquid phase chromatograph from Waters Alliance 2,695 Separation Module (quaternary pump system, degasser, autosampler, column oven, all coupled to a network of Waters 996 PDA diode detectors) (Waters, Ireland); LiChrospher 100 RP18 column from Merck (LiChrospher 100 RP-18 (5 μm)) (Cambridge, MA, USA); MicroMass Quattro Micro equipped with ESI source in positive and negative mode (Waters, Ireland); Christ Alpha 1–4 freeze dryer; microplate reader from FLUOstar BMGLabtech (Ortenberg, Germany). Mettler Toledo scale (Columbus, USA); 04644-Series Digital stirring hotplate from Cole Parmer (Vernon Hills, IL, USA); Heraeus Biofuge Stratos centrifuge from Kendro Laboratory Products (Osterode, Germany); Hitachi U-2000 spectrophotometer (Tokyo, Japan); Delsa Nano C (Coulter, CA, USA). Contour next glucometer (Lisbon, Portugal); Ugo Basile 7,140 plethysmometer (Gemonio, Italy); Shimadzu Libror EB-2800 scale (Kyoto, Japan); Janetzki T 32 centrifuge (Wallhausen, Germany).

### 2.3 Methods

#### 2.3.1 Extract preparation

In the preparation of extracts, fresh samples of dragon fruit (*Hylocereus costaricensis*) provided by a Portuguese producer (Atlantis Cactus Park, Sesimbra, Portugal) were used.

The procedure was performed according to the methods referred to in the literature (15, 42) with some changes. In the preparation of extracts, two different solvents were used (ethanol and distilled water) to evaluate which of these were the best extractant.

The ethanol extraction was prepared according to Macias-Ceja et al. (42) with certain modifications, referred below. A fresh fruit without skin, was crushed in a blender and mixed with an ethanol:water solution (50:50, v/v) at room temperature. The obtained mixture was centrifuged at 880 × g for 20 min, and the supernatant was filtered using compresses. This step was repeated 4 times. Finally, the filtered extract was concentrated at 55 °C, and the concentrated extract was stored at 4 °C. With this methodology, two extracts were prepared in a ratio of 1:1 (w/v) and 1:2 (w/v). 50.67 g was used for the 1:1 and 50.04 g of fresh fruit for the 1:2 preparation.

Aqueous extracts were obtained using the method of Al-Radadi (15) with some modifications, which are described below. Dragon fruit, a fresh fruit without the skin, was mixed with distilled water and processed in a blender at a ratio of 1:2 (w/v), using 130 g of dragon fruit and 260 mL of distilled water. The mixture was then placed in tubes and centrifuged at 880 ×*g* for 20 min. Subsequently, the supernatant was filtered using compresses, repeating the procedure 3 times the centrifugation and filtration step. The filtrate was stored at −20 °C.

The extracts obtained were characterized by determining the content of total phenols and total flavonoids. The results were then compared, and the solvent and methodology were selected to obtain an extract richer in phenolic compounds.

After selecting the best extraction conditions, an extract was prepared with the remaining sample (811.1 g of dragon fruit). Finally, the extract was concentrated in an oven at 100 °C for 150 min to obtain an extract richer in phenolic compounds.

The extract was analyzed for total phenols, total flavonoids, and key phenolic compounds.

##### 2.3.1.1 Extract characterization

###### 2.3.1.1.1 Quantification of total phenolic content

The total phenolic content was determined using the method of Direito et al. (43). In a tube, 100 μL of the sample was added with 200 μL of Folin–Ciocalteu reagent (diluted in water 1:10, v/v). The sample was left to react for 3 min, and 1 mL of sodium carbonate (15% w/v) and 2 mL of deionized water was added. After 1 h, the absorbance was measured at 765 nm (Hitachi L-2000, Hitachi High Technology, Tokyo, Japan) against the blank. Gallic acid was used as a standard at concentrations between 10 and 400 mg/L to obtain a calibration curve. The results were expressed in milligrams of gallic acid equivalents (mg GAE) per 100 g of fresh fruit (FF) and per 100 mL of extract. Determinations were made in triplicate, and results were expressed as mean ± SD.

###### 2.3.1.1.2 Quantification of total flavonoid content

Total flavonoid content was measured using the method of Çam et al. (44). In a 10 mL flask, 1 mL of sample was added, 4 mL of distilled water, and 300 μL of sodium nitrite (5% w/v). After 5 min, 300 μL of aluminum chloride (10% w/v) was added and allowed to react for 6 min. Subsequently, 2 mL of sodium hydroxide (1 M) was added, and the volume was made up to 10 mL with deionized water. The absorbance was measured at 510 nm against the blank. Catechin was used as standard at concentrations of 20 to 150 mg/L. The results were expressed in mg of catechin equivalents (mg EC) per 100 g of fresh fruit (FF) and per 100 mL of extract. Determinations were made in triplicate, and results were expressed as mean ± SD.

###### 2.3.1.1.3 Phytochemical characterization of the extract by LC-DAD-ESI-QqQ-MS/MS

Analysis of the extract by LC-DAD-ESI-QqQ-MS/MS was carried out at the FFUL Structural Analysis Laboratory. The equipment used was a liquid phase chromatograph with a LiChrosper column (5 mm, 250 × 4 mm; Merck, USA) coupled to a diode network. At Tandem mass spectroscopy a triple quadrupole type mass spectrometer and an electrospray ion source (ESI) were used. In HPLC, the separation of compounds was carried out by reversed-phase chromatography, at 35 °C, using a mobile phase consisting of a gradient of solvents: (A) 0.1% (v/v) formic acid (HCOOH) in water and (B) acetonitrile with a flow rate of 300 μL/min. The injection volume was 20 μL and the diode array (DAD) was used at a wavelength of 210 to 700 nm. The gradient was for eluent A and B, respectively: at 0 and 10 min 95 and 5%; at 30 min 82 and 18%; at 44 and 64 min 64 and 36%; 90 and 100 min 10 and 90%; 101 and 120 min 95 and 5%.

In tandem mass spectroscopy (MS/MS), the electrospray source, in positive (ESI+) or negative (ESI-) ionization mode, was maintained at 120 °C. A capillary potential of 2.5 kV and a source potential of 30 V were applied. Data acquisition occurred in “Full Scan” mode in the range m/z 60–1,100. MS/MS conditions were optimized when standard solutions were available by performing analysis in multiple reaction monitoring (MRM) mode to obtain greater selectivity and sensitivity. High-purity nitrogen was used as drying and nebulizing gas. The MassLynx program version 4.1 was used in data acquisition and processing, and compound identification was carried out based on comparison with commercial standard solutions available in the laboratory and with data referenced in the literature.

#### 2.3.2 Phytosomes preparation

The phytosomes were prepared according to the method of Direito et al. (43). Dragon fruit extract was added to phosphatidylcholine (1:1, molar ratio) dissolved in 20 mL of ethanol. Then, the mixture was heated to 25 °C with a magnetic stirrer at 300 rpm for 2 h. Finally, 40 mL of 2% acetic acid was added, and the mixture remained in the same condition as before for 24 h. The phytosomal formulation was stored at 4 °C.

##### 2.3.2.1 Physical characterization of phytosomes

The phytosomes were characterized according to the method described by Direito et al. (43). The phytosomes were diluted with distilled water, and the average particle size, polydispersity index (PI), and zeta potential (ζP) were analyzed on the Delsa Nano C (Coulter, CA, USA), at room temperature (25 °C).

##### 2.3.2.2 Determination of encapsulation efficiency (EE)

The determination of EE was determined by evaluating the non-encapsulated extract fraction according to the method described by Direito et al. (43). In a tube, 2 mL of the phytosomal formulation was added to 2 mL of sunflower oil and homogenized by agitation. The mixture was centrifuged at 15000 × g for 30 min at 4 °C. Subsequently, supernatant (corresponding to non-encapsulated phenolic compounds) from the phytosomal preparation was separated for analysis. Encapsulation efficiency was determined through analysis.

Using the Folin–Ciocalteu method to quantify the total phenol content, following the method of Direito et al. (43), in a tube, the supernatant sample (100 μL) was added to 200 μL of Folin–Ciocalteu reagent, after 3 min, 1 mL of sodium carbonate (15% w/v) and 2 mL of deionized water were added, after 1 h (in the dark at room temperature), the absorbance was measured at 765 nm against the blank. A curve of gallic acid calibration was constructed, using concentrations between 10 and 300 mg/L. Results were expressed in milligrams of gallic acid equivalents (mg GAE) per 100 mL. Determinations were made in triplicate, and the results were expressed as mean ± SD. The encapsulation efficiency (EE, %) was estimated through the difference between the content of total phenolic compounds (TPC) in the initial extract and the content of total phenolic compounds present in the supernatant (fraction of non-encapsulated extract), applying Equation 1.


(1) $$\mathrm{EE}\;(\%)=\frac{\begin{array}{c} \mathrm{TPC}\;(\text{initial extract})-\mathrm{TPC}\\ \;(\text{free extract in supernatant}) \end{array}}{\mathrm{TPC}\;(\text{initial extract})}x100\;$$

##### 2.3.2.3 Determination of the antioxidant activity

Antioxidant activity was evaluated using the method described by Mota et al. (45) with modifications. In cuvettes, 10 μL of sample was mixed with 990 μL of DPPH solution and allowed to react for 30 min in the dark at room temperature. Quercetin (10 mg/mL) was used as a positive control. A sample containing 10 μL of deionized water and 990 μL of DPPH was used as an absorbance control. Absorbance was measured at 517 nm against a 70% ethanol blank using a UV–Visible Spectrophotometer (Hitachi L-2000 from Hitachi High Technology, Tokyo, Japan). Analyses were performed in triplicate. DPPH sequestration capacity was calculated using Equation 2:


(2) $$\text{Scavenging activity}\;(\%)=\frac{\begin{array}{c} \text{Absorbance control}\\ -\text{Absorbance sample} \end{array}}{\text{Absorbance control}}\times 100\;$$

#### 2.3.3 Animals

In *in vivo* models, male Wistar rats (*n* = 24) weighing between 115 g and 205 g were used. Before the experimental tests, the animals underwent a period of acclimatization in the animal facilities of the Faculty of Pharmacy of the University of Lisbon (FFUL). For a week, the animals were kept at a temperature of 21 °C, with daily 12-h light cycles and free access to water and food. Experiments were performed according to the animal welfare organ of the Faculty of Pharmacy, University Lisbon, approved by the competent national authority (*Direção-Geral de Alimentação e Veterinária*—DGAV) under the project “*Abordagem Sustentável, Segura e eScalável (3S) para a valorização do licopeno: conduzindo o conhecimento para uma interface academia-indústria para o tratamento tópico da inflamação*” and in accordance with the EU Directive (2010/63/EU), the Portuguese laws (DL 113/2013, 2,880/2015, 260/2016 and 1/2019) and all relevant legislation, for the use and care of animals in research.

##### 2.3.3.1 Sugar overload model

To evaluate the antihyperglycemic activity of the extract and phytosomes, the sugar overload model was used. Metformin was used as an anti-diabetic to compare the results obtained. The animals were administered with amounts of extract and phytosomes that corresponded to a dose of 5 and 2.3 mg TPC/kg of animal, respectively. 24 animals were weighed and randomly divided and distributed among the 4 experimental groups. The study included four groups (*n* = 6 each): a Control group with unrestricted access to food and water and no treatment; a Metformin group given 300 mg/kg metformin for 14 days; a Dragon fruit extract group pre-treated with 5 mg GAE/kg dragon fruit extract for 14 days; and a Phytosome group pre-treated with 2.3 mg GAE/kg dragon fruit extract-loaded phytosomes for 14 days.

Samples were administered to the animals by gavage daily for 14 days. During this period, the animals had free access to water and food, except for the day before the test where they only had free access to water. On the night of the 14th day, the animals fasted. On the 15th day, glycemia was measured in all animals and then they were administered with a glucose solution (2 g/kg) by oral gavage to induce hyperglycemia.

The used sugar overload method was based in Grácio et al. (46), with some changes described below. On the 15th day of the trial, all animals were pricked on the tail and a drop of blood was collected to measure their basal blood glucose level (G0) using a glucometer, and then the animals were administered with a glucose solution at 0 min. Then, a solution of glucose (2 g/kg), was administered by oral gavage. After 30, 60 and 120 min (G30, G60, and G120, respectively) of glucose administration, the animals’ glucose levels were measured again. The increase in blood glucose levels was determined as described in Equation 3.


(3) $$\text{Increased blood sugar levels}\;(\%)=\frac{G30,60\;\text{or}\;120-G0\;}{G0\;}\times 100\;$$

At the end of the test, 2 animals from each group were anesthetized with a mixture of ketamine 80 mg/kg and xylazine 8 mg/kg, and blood was collected by cardiac puncture to determine the biomarkers present in the serum. The collected blood was allowed to clot and centrifuged at 880 × g for 20 min. The serum was then separated and frozen for future biomarker analysis.

##### 2.3.3.2 Paw edema model induced by *λ*-carrageenan

The 24 animals used in this model were weighed and randomly divided and distributed into the following groups: Negative control group (*n* = 6)—the animals had no pre-treatment and on the 15th day they were injected with 0.1 mL of sterile saline in the left hind paw (subplantar injection); Positive control group (carrageenan) (*n* = 6)—the animals had no pre-treatment and on the 15th day paw edema was induced by subplantar injection into the left hind paw of 0.1 mL of a 1% *λ* dispersion -carrageenan in saline solution; Dragon fruit extract group (*n* = 6)—the animals were pre-treated with dragon fruit extract (5 mg GAE/kg) for 14 days and on the 15th day paw edema was induced by subplantar injection in the left hind paw 0.1 mL of a 1% dispersion of *λ*-carrageenan in saline solution; Phytosomes loaded with dragon fruit extract (*n* = 6)—animals were pre-treated with phytosomes (2.3 mg GAE/kg) for 14 days and on the 15th day paw edema was induced by subplantar injection in the hind paw left of 0.1 mL of a 1% dispersion of λ-carrageenan in sterile saline solution.

The administration of samples to the animals by oral gavage was carried out daily for 14 days. During this period, the animals had access to water and food.

After 14 days, the volume of the left hind paw of each animal (from all groups) was measured in a plethysmometer before carrageenan injection (V0). The animals were then anesthetized with an injection of a mixture of ketamine 75 mg/kg + medetomidine 0.50 mg/kg (intraperitoneally) so that the animals did not feel the pain of inducing paw edema during the test. Then, 0.1 mL of a sterile saline solution with 1% λ-carrageenan was injected into the hind paw from the left side of each rat, except for the negative control group, which was injected with 0.1 mL of a sterile saline solution. The paw volume of all groups was measured again 3 and 6 h after the carrageenan injection (V3 and V6, respectively). Increased paw volume (edema) was determined as described by Equation 4.


(4) $$\mathrm{Paw}\;e\text{dema}\;(\%)=\frac{V3\;\text{or}\;6-V0}{V0}\times 100\;$$

At the end of the test, 2 animals from each group were anesthetized with isoflurane and blood was collected by cardiac puncture to determine the biomarkers present in the serum. The collected blood was allowed to clot and centrifuged at 970 × g for 20 min. The serum was then separated and frozen for future analysis of serum markers of inflammation.

### 2.4 Evaluation of changes in organs function

Inflammatory markers, including C-reactive protein (CRP) and the cytokine IL-6, were measured. Additionally, kidney biomarkers (albumin, urea, and creatinine) and liver biomarkers (alkaline phosphatase (ALP) alanine transferase (ALT), aspartate aminotransferase (AST), gamma-glutamyltransferase (GGT) and total proteins) were assessed.

The IL-6 concentration was measured using a high-sensitivity immunoenzymatic ELISA method using the Quantikine HS ELISA Kit from R&D Systems (Abingdon, UK), according to the manufacturer’s instructions. The IL-6 concentration was expressed in pg./mL.

CRP (mg/dL), total proteins (g/dL), albumin (g/dL), urea (mg/dL), creatinine (mg/dL), AST (U/L), ALT (U/L), ALP (U/L) and GGT(U/L) in rat serum were determined using kits according to the manufacture instructions Roche Diagnostics GmbH (Mannheim, Germany), on the automatic analyzer Cobas Analyzer Roche® by Roche Portugal (Amadora, Portugal).

### 2.5 Statistical analysis and experimental design

The statistical analysis of the results was carried out using GraphPad Prism 10. The experimental design incorporated multiple replicates to ensure robustness and reliability of the results. For all experiments, statistical factors (such as treatment type and dose levels) and response variables were clearly defined to facilitate the interpretation of the findings.

#### 2.5.1 Spectrophotometric methods

The statistical treatment involved determining the mean and standard deviation (SD) for triplicate measurements for each experimental condition. Differences between sample means were evaluated using ANOVA (analysis of variance), followed by the Tukey parametric test. Response variables included antioxidant activity (percentage inhibition) measured across varying concentrations of samples. A *p*-value <0.05 was considered statistically significant, indicating statistically different values.

#### 2.5.2 Physical characterization of phytosomes and EE (%)

Results for the physical characterization of phytosomes and encapsulation efficiency (EE) were based on triplicate measurements. The mean and standard deviation were determined, and statistical significance was assessed using ANOVA. The response variables in these analyses included particle size, polydispersity index (PDI), zeta potential, and encapsulation efficiency. A p-value <0.05 was considered statistically significant.

#### 2.5.3 DPPH method

For antioxidant activity determined using the DPPH method, triplicate samples were used for each test condition to ensure reproducibility. The mean and standard deviation were calculated, followed by ANOVA to evaluate differences between sample means. The Tukey parametric test was used for post-hoc comparisons. The response variable was the percentage of radical scavenging activity (% inhibition) at different concentrations. A *p*-value <0.05 indicated statistical significance.

#### 2.5.4 *In vivo* studies

The *in vivo* experimental design included six animals per group to provide sufficient statistical power. Blood glucose levels were measured at four time points (0, 30, 60, and 120 min) for each group. The statistical treatment involved calculating the mean and standard deviation, followed by ANOVA to evaluate differences between group means. The Bonferroni post-hoc test was applied for pairwise comparisons. The response variables included blood glucose levels and the area under the curve (AUC) for blood glucose over time. A *p*-value <0.05 was considered statistically significant.

Across all experiments, statistical factors such as treatment type (e.g., control, metformin, extract, or phytosomes formulations), dose levels, and time points were considered to account for variability and ensure reliable comparisons. All analyses incorporated replicates to improve the robustness and reliability of the findings.

## 3 Results

### 3.1 Extract characterization

#### 3.1.1 Quantification of total phenolic content

The study compared methods for measuring total phenols and flavonoids in extracts using distilled water and 50% ethanol as solvents. The Folin–Ciocalteu method with gallic acid as a standard (linear from 10 to 300 mg/L, *R*^2^ = 0.9959 and 0.9912) assessed total phenolic content, as detailed in Table A1. Results showed significant differences (*p* < 0.0005) among extracts; water-extracted samples had the highest total phenol content at 165.8 ± 0.895 mg GAE/100 g FF.

#### 3.1.2 Quantification of total flavonoid content

The calibration curves were constructed using catechin as a standard in concentrations between 20 and 150 mg/L, and absorbance was measured at 510 nm. Table A2 contains the corresponding data.

The values obtained present statistically significant differences between the solvents used (water and 50% ethanol) and the sample preparation proportions (1:1 and 1:2), Table A2. The aqueous extract had the highest total flavonoid content (110.1 mg EC/100 g FF), while the 1:2 ethanolic extract contained 22.447 mg EC/100 g FF.

Thus, water proved to be a better extracting solvent, obtaining the extract richest in phenolic compounds, which is why the methodology referring to this solvent was used for the remaining fresh fruit sample. In this study 811.1 g of dragon fruit (fresh fruit) were applied with distilled water (1:2, w/v) for extract preparation. The data relating to the calibration curves of total phenols and flavonoids determinations for the aqueous extract for the rest of this study is found in Table A3.

The aqueous extract of dragon fruit prepared for *in vitro* and *in vivo* application in this study has a total phenol content of 41.037 mg GAE/100 mL and a total flavonoid content of 13.930 mg CE/100 mL. This extract was distributed into falcon tubes and stored at −20 °C for future analysis.

#### 3.1.3 Phytochemical characterization of the extract by LC-DAD-ESI-QqQ-MS/MS

Pitahaya phenolic extract was analyzed using LC-DAD-ESI-QqQ-MS/MS. Chromatograms at 280 nm (for phenols) and 532 nm (for betacyanins) were compared, as shown in Figure A1 (47). Comparing the chromatographic profiles of the sample with mixtures of standards analyzed under the same conditions, it was possible to identify some compounds, as can be seen in Table 1 and Figures A2–A7.

**Table 1:** Possible identification of phytochemicals by MS/MS in dragon fruit extract.

RT (min)	UV λmax (nm)	[M − H] − m/z	[M + H] + m/z	Possible identification	References
8.73	280	191	–	Citric acid	(48)
12.36	280	117	–	Succinic acid	(48)
26	532	–	551	Betanin	(47, 51, 52)
				Isobetanin	
31.28	532	–	637	Phyllocactin	(47, 52, 53)
				Isophyllocactin	
				Phyllocactin II	
				Betanidin-5-O-(6′-O-3-hydroxybutyryl)-β-glucoside	
46.30	280	–	595	Cyanidin-3-o-rutinoside	(49)
47.21	280	609	–	Rutin (Quercetin-3-O-rutinoside)	(47)
49.86	280	–	195	Ferulic acid	(50)

Retention time (RT), maximum absorption wavelength, and molecular ion.

In the dragon fruit extract, it was possible to identify several types of compounds, namely organic acids and phenolic compounds. Citric acid and succinic acid are organic acids, and cyanidin-3-O-rutinoside (anthocyanidin, which is a type of flavonoid), ferulic acid (phenolic acid), and rutin (flavonol which is a type of flavonoid) are phenolic compounds (47–50).

It was not possible to identify all the compounds present in the extract, so literature was used for possible identification. The compound with protonated molecular ion m/z 551 was identified in dragon fruit extracts by other authors as betanin or isobetanin and the compound with molecular ion m/z 637 as phylocactin, isophylocactin, phylocactin II or betanidin-5-O-(6′-O-3-hydroxybutyryl)-*β*-glucoside (47, 51–53).

The phenolic compounds cyanidin-3-o-rutinoside, ferulic acid, and rutin have been associated in previous studies with dragon fruit’s antioxidant, anti-hyperglycemic, and anti-inflammatory potential. These activities will be the focus of the next stages of this work.

### 3.2 Physical characterization of phytosomes—dynamic laser scattering (DLS), laser of light

The physical characterization of phytosomes was done by Dynamic Laser Scattering (DLS) and the size of the nanoparticles obtained was 1,329 ± 121.0 nm (greater than 1,000 nm,) suggests that they are not suitable for intestinal absorption and, consequently, there is a low bioavailability of the encapsulated phytocomponents (54, 55). Therefore, the >1 μm mean diameter is a methodological limitation, and for future work practical downsizing strategies will be proposed.

The polydispersity index (PI) value > 0.3 is indicative of a polydisperse system, i.e., the particle size distribution is very broad (56, 57). A polydisperse system has a greater tendency to aggregate than a monodisperse system (56). The phytosomes in this work presented a PI of 0.633 ± 0.039 (> 0.3) which is indicative of low uniformity (56, 57).

Zeta potential (ζ*P*) is an indicator of dispersion stability against particle aggregation or deposition. Zeta potential values above the ±30 mV range trigger greater electrostatic repulsive forces, preventing particle aggregation and keeping the system stable (58, 59). Nanoparticles with a zeta potential between ±10 are considered neutral (59). In this work, the phytosomes presented a ζ*P* value of −16 ± 1 mV, which means that there was aggregation of particles and that the system is not homogenous, according to the literature (58, 60). Future work will develop practical strategies to overcome the morphologic nanocarriers limitations.

#### 3.2.1 Determination of encapsulation efficiency (EE)

The determination of the content of phenolic compounds was carried out using the Folin–Ciocalteu method. The calibration curve was drawn using gallic acid as a standard in concentrations between 10 and 300 mg/L, with an equation y = 0.0021x + 0.0368 and *R*^2^ = 0.9908. The total phenolic content of the phytosomal formulation was determined: in the concentrated extract with a result of 41.037 ± 0.257 mg GAE/100 mL, and in the supernatant with a result of 22.160 ± 1.533 mg GAE/100 mL.

Applying Equation 1, the encapsulation efficiency was 46%, which means that only part of the extract was encapsulated.

### 3.3 Determination of antioxidant activity (DPPH)

The results of the determination of antioxidant activity are described in Table A4.

The results of this study demonstrate that the prepared phytosomes have a greater antioxidant activity than the extract. It is also verified that the DPPH• radical scavenging activity of both is very similar, extract has 33.4 ± 0.4%, and phytosomes have 38.3 ± 0.8%, which means that phytosomes with a lower concentration (18.877 mg GAE/mL) than the extract (41.037 mg GAE/mL) can have the same effect, allowing better control of the dose used.

The findings suggest that quercetin exhibits superior DPPH• scavenging activity compared to both the phytosomal formulation and the dragon fruit extract. These results align with those reported by Mahdi et al. (60), who found that the positive control (gallic acid) demonstrated higher antioxidant activity (using the DPPH method) than the *H. undatus* extract and its gold nanoparticle formulation.

### 3.4 Sugar overload model

The antihyperglycemic activity was evaluated at a dose of 5 mg GAE/Kg of dragon fruit extract and a dose of 2.3 mg GAE/kg for phytosomes. The results obtained can be observed in Tables A5 and A6.

Animals in the control group experienced an increase in blood glucose levels at 30 min, followed by a decrease at 60 min. However, even after 120 min, the levels had not returned to baseline values.

In the metformin group (300 mg/kg), blood glucose levels increased significantly 30 min after glucose loading, though the increase was less pronounced than in the control group, indicating metformin’s anti-glycemic effect. Blood glucose levels began to decrease at 60 min and continued for 120 min, although the values were similar to those of the control group by this point. Metformin, a common medication for type 2 diabetes, effectively controlled the glycemic peak at 30 min but did not show significant effects at 60 and 120 min.

The group treated with a low dose (5 mg GAE/kg) of dragon fruit extract showed no statistically significant differences compared to the control group, suggesting that the dose was insufficient to exert an antihyperglycemic effect.

The group treated with phytosomes (2.3 mg GAE/kg) demonstrated an antihyperglycemic effect at 30, 60, and 120 min compared to the control group. This indicates that phytosomes containing pitahaya extract effectively controlled blood glucose levels. Phytosomes containing dragon fruit extract demonstrated a superior anti-hyperglycemic effect compared to the extract alone, even with less than half the concentration of phenolic compounds (46%). This suggests that using phytosomes is an effective strategy to overcome the low bioavailability of these compounds. When comparing the phytosome group to the metformin group, similar glycemic control was observed at 30 and 60 min, with the phytosome group achieving even lower glycemic levels than metformin at 120 min.

### 3.5 Paw edema model induced by *λ*-carrageenan

**Figure 1:**
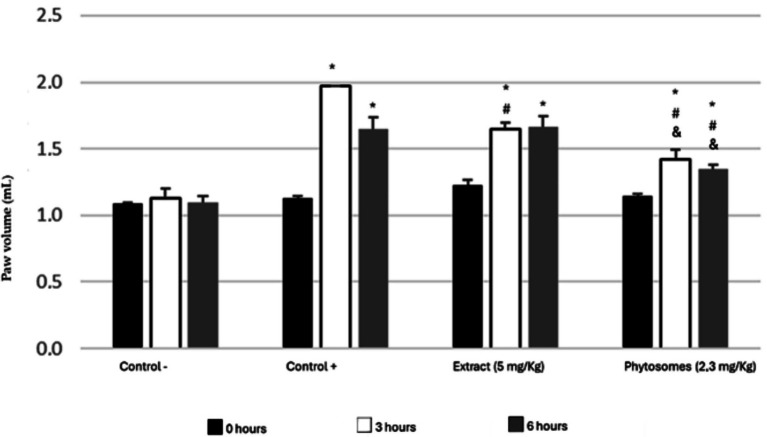
Paw volume induced by carrageenan after 14 days of treatment with dragon fruit extract (5 mg/kg) and phytosomes (2.3 mg/kg). Four groups of rats were tested: negative control (without edema induction), positive control (with edema induction), extract (5 mg/kg), and phytosomes (2.3 mg/kg). * *p* < 0.001 vs. control (−); # *p* < 0.01 vs. control (+); & *p* < 0.05 vs. extract.

The anti-inflammatory activity was evaluated for a dose of 5 mg GAE/kg of dragon fruit extract and a dose of 2.3 mg GAE/kg of phytosomes. The results obtained are presented in Table A7 and Figure 1.

The paw volume of the negative control group should have remained constant, as paw edema was not induced with carrageenan. Small variations can be explained by the movements of the animals at the time of measurement. The paw volume at 0, 3, and 6 h was 1.10 ± 0.01 mL, 1.13 ± 0.07 mL, and 1.09 ± 0.05 mL, respectively, with no statistically significant differences (*p* > 0.2).

All groups, except for the negative control group, developed edema due to the carrageenan injection, validating the model’s effectiveness. In the positive control group, the initial paw volume was the same as the negative control (1.10 ± 0.02 mL). Following carrageenan administration, paw volume increased to 1.97 mL (75.11%) after 3 h. At the 6-h mark, the paw remained inflamed, measuring 1.65 ± 0.09 mL.

The group treated with a 5 mg GAE/kg dose of dragon fruit phenolic extract showed an increase in paw volume 3 h after carrageenan injection, with a slight further increase at 6 h, indicating that inflammation persisted. Initially, the paw volume in this group was similar to the positive control, but it was significantly lower at 3 h (*p* < 0.01). However, by 6 h, the volume was comparable to the positive control. These results suggest that the dragon fruit extract reduced edema at 3 h but was ineffective at 6 h.

Animals treated with phytosomes showed a significant decrease in paw volume at 30 and 60 min compared to the positive control group. Additionally, phytosomes containing a lower dose of phenolic compounds (2.3 mg/kg) prevented the inflammation from reaching the peaks observed in both the positive control and extract-treated groups at 3- and 6-h post-edema induction (24.13 and 17.4%, respectively).

Inflammatory cytokines, C-Reactive protein (CRP), and IL-6, were also determined in the animals’ serum after the test. The results obtained are presented in Table 2.

**Table 2:** Inflammatory cytokines determined in the serum of animals after the anti-inflammatory assay.

Groups	Inflammatory cytokines	
	CRP (mg/dL)	IL-6 (pg/mL)
Control −	0.008 ± 0.006	1.500 ± 0.000
Control +	0.014 ± 0.015	2.285 ± 1.110
Extract (5 mg/kg)	0.009 ± 0.010	2.065 ± 0.799
Phytosomes (2.3 mg/kg)	0.011 ± 0.002*	1.500 ± 0.000*

*p < 0.05 vs. Control +.

C-Reactive protein levels were similar across all groups, indicating a slight acute inflammatory reaction. The group treated with phytosomes had a value of 0.011 ± 0.002 mg/dL, comparable to the positive control group treated with carrageenan (0.014 ± 0.015 mg/dL) and the negative control group (0.008 ± 0.006 mg/dL), with no significant differences observed between the control groups. Additionally, the extract group had a value of 0.009 ± 0.010 mg/dL, which is also similar to the negative control group.

Regarding IL-6 cytokine levels, the phytosome-treated group showed the same value as the negative control group that did not receive carrageenan (1.500 pg./mL), indicating anti-inflammatory activity. Phytosome treatment restored IL-6 to basal values (1.5 pg./mL) and held CRP at control levels (Table 2). This cytokine attenuation, combined with the paw-edema outcome, points to early NF-κB pathway modulation by key constituents (cyanidin-3-O-rutinoside, betanin, ferulic acid).

In contrast, the extract-treated group had values (2.065 ± 0.799 pg./mL) similar to the positive control group that received carrageenan (2.285 ± 1.110 pg./mL), suggesting minimal anti-inflammatory activity. These values corroborate the earlier results, as they do not indicate acute inflammation at the end of the test. Overall, the values between all groups are statistically equivalent.

### 3.6 Evaluation of changes in organs function

The animals’ sera were analyzed to determine kidney biomarkers (albumin, urea, and creatinine) and liver biomarkers (AST, ALT, ALP, and GGT). These biomarkers help to assess whether the dragon fruit extract and phytosomes caused any damage to the kidneys and liver.

Table 3 presents the biomarker values for animals in the anti-hyperglycemic activity test, while Table 4 shows the values for the anti-inflammatory activity test. The results from both tests demonstrated that the dragon fruit extract and phytosomes were not toxic to the animals, as the organ control biomarkers in their serum were within reference values (61–69).

**Table 3:** Biochemical markers determined in animal sera after studying anti-hyperglycemic activity.

Biochemical markers	Control	Extract (5 mg/kg)	Phytosomes (2.3 mg/kg)	Reference Interval
Albumin (g/dL)	4.300 ± 0.141	4.355 ± 0.233	4.080 ± 0.453	2.5–4.8
ALP (U/L)	172.0 ± 14.142	166.5 ± 19.092	220.5 ± 61.518	166.0 ± 7.1–290 ± 63
ALT (U/L)	46.500 ± 21.920	20.500 ± 0.707	21.000 ± 2.828	20.0–61.0 ± 2.4
AST (U/L)	247.0 ± 111.723	181.0 ± 83.439	173.5 ± 91.217	23.0–187.0 ± 102.0
Creatinine (mg/dL)	0.278 ± 0.029	0.235 ± 0.016	0.258 ± 0.045	0.2–0.8
GGT (U/L)	<0.100 ± 0.000	<0.100 ± 0.000	<0.100 ± 0.000	0.0
Total proteins (g/dL)	5.900 ± 0.000	6.005 ± 0.474	5.850 ± 0.495	4.0–8.6
Urea (mg/dL)	29.000 ± 4.243	25.000 ± 1.414	22.500 ± 0.707	12–61.7 ± 1.6

The results were expressed as mean ± SD.

**Table 4:** Biochemical markers determined in animal sera after studying anti-inflammatory activity.

Biochemical markers	Negative control	Positive control	Extract (5 mg/kg)	Phytosomes (2.3 mg/kg)	Reference Interval
Albumin (g/dL)	3.937 ± 0.297	4.035 ± 0.092	4.500 ± 0.226	4.160 ± 0.042	2.5–4.8
ALP (U/L)	198.0 ± 27.622	166.5 ± 12.021	196.0 ± 38.184	167.0 ± 0.000	166.0 ± 7.1–290.0 ± 63
ALT (U/L)	47.000 ± 9.000	105.5 ± 70.004	33.500 ± 9.192	35.000 ± 4.243	20.0–61.0 ± 2.4
AST (U/L)	208.3 ± 69.060	497.0 ± 323.9	162.0 ± 83.853	160.0 ± 36.770	23.0–187.0 ± 102.0
Creatinine (mg/dL)	0.252 ± 0.053	0.328 ± 0.001	0.342 ± 0.076	0.235 ± 0.026	0.2–0.8
GGT (U/L)	<0.100 ± 0.000	<0.100 ± 0.000	<0.100 ± 0.000	<0.100 ± 0.000	0.0
Total proteins (g/dL)	5.540 ± 0.378	5.930 ± 0.467	6.330 ± 0.424	5.715 ± 0.021	4.0–8.6
Urea (mg/dL)	24.000 ± 5.292	27.500 ± 4.950	24.500 ± 0.707	23.000 ± 1.414	12–61.7 ± 1.6

The results were expressed as mean ± SD.

Oral administration of the extract and phytosomes did not alter the levels of organ control biomarkers in the animals’ serum, remaining at normal levels, indicating that the extract and phytosomes did not present any toxicity effect on animals.

## 4 Discussion

Abd Manan et al. (70) prepared an aqueous extract of *Hylocereus polyrhizus* under conditions similar to this work and obtained a value of 25.27 mg GAE/100 mL for TPC, which is very close to the value obtained in this study, 22.933 mg GAE/100 mL. Abirami et al. studied the differences between three species of dragon fruit (H*ylocereus* spp.), with the *H. costaricensis* extract having a higher content of total phenols (130.0 mg GAE/100 g FF) and which is below the value obtained in this work (165.8 mg GAE/100 g FF) (4). The results obtained were different from Sulaiman et al., as they obtained higher TPC concentration for the ethanolic extract (1.395 mg GAE/g FF) than for the aqueous extract (1.130 mg GAE/g FF) of red-fleshed dragon fruit. In addition to ethanol being the best extracting solvent, this extract presented lower values than the aqueous extract obtained in this work (1.658 mg GAE/g FF) (71). The difference in total phenolic content in the two studies can be explained by different soil and climate conditions and different species (72).

The LC-DAD-ESI-QqQ-MS/MS method allowed us to identify compounds such as citric acid, succinic acid, ferulic acid, cyanidin-3-o-rutinoside, rutin, betanin or isobetanin (m/z 351), phylocactin, isophylocactin, phylocactin II or betanidin-5-O-(6′-O-3-hydroxybutyryl)-*β*-glucoside for the molecular ion m/z 367 (47, 51–53). Osorio-Esquivel et al. also identified the compound with molecular ion m/z 551 as betanin or isobetanin in fruits of the *Opuntia joconostle* cactus. The compound with molecular ion m/z 637 was identified as phylocactin or unknown betacyanin (52). High-Resolution Mass Spectrometry may be used in future studies to identify minor isobaric constituents.

Considering authors knowledge, this study presents the first physical characterization of phytosomes containing dragon fruit extract. Previous studies have prepared and characterized phytosomes with various plant extracts, showing similarities to the results of this study (73–75).

Dhase et al. prepared phytosomes using bael (*Aegle marmelos*), also known as golden apple, leaf extract with a cholesterol to phosphatidylcholine ratio of 1.5:5.5, resulting in nanoparticles with a size of 1,570 nm and a polydispersity index (PI) of 0.456. Both the nanoparticles in this study and those from Dhase et al. exceed 1,000 nm in size, which can influence absorption, and have a PI greater than 0.3, indicating a polydisperse system (73).

Similarly, Jagtap et al. prepared phytosomes with maidenhair extract (*Adiantum capillus-veneris*) achieving a nanoparticle size of 1,045 nm, which is slightly smaller than present results but comparable. In their study, they also prepared phytosomes with a zeta potential of −17.22 mV, which is similar to present findings (74).

Surini et al. (75) formulated phytosomes with grape seed extract (*Vitis vinifera* L) achieving a zeta potential of −25.2 mV. This value falls within the ±30 mV range, like the zeta potential of prepared phytosomes in this study. The encapsulation efficiency observed in this study is lower than that reported by other researchers for different plant extracts in phytosomes (43, 76–78).

The novel findings of this study highlight the need for further optimization to improve encapsulation efficiency as well as homogeneity, and particle size diameter of the formulation. Even with those limitations this nanosystem demonstrated higher bioactivity than free extract. Notably, this study is the first to successfully encapsulate dragon fruit extract in a phytosomal formulation, representing a significant milestone in green encapsulation technology research.

Encapsulation improves the bioavailability and stability of phenolic compounds, overcoming the challenges of their rapid degradation and poor absorption when used in their free form. This method allows for precise dosing, enabling therapeutic effects at lower concentrations, as evidenced by the enhanced antioxidant, antihyperglycemic, and anti-inflammatory activities observed with phytosomes compared to free extracts, potentially enhancing the reliability and consistency of their health benefits.

Furthermore, this approach aligns with growing trends in functional foods and nutraceuticals, offering a natural and innovative means of maximizing ‘s health-promoting properties. These advantages underscore the potential of nanoparticle systems to revolutionize how bioactive compounds are utilized and applied.

This study represents the first known attempt to prepare phytosomes with dragon fruit extract and assess their antioxidant activity.

Abirami et al. (4) analyzed three species of dragon fruit (*Hylocereus* spp.) for their biochemical and morphological properties, revealing that the extract of *H. costaricensis* exhibited a DPPH• scavenging activity of 36.0%, closely matching the 33.365% observed in this study. Phytosomes prepared with other vegetal extracts obtain similar antioxidant activities, like phytosomes with *Bombax ceiba* leaf extract achieving a DPPH• radical inhibition of 42.939% (79). This antioxidant activity is like the phytosomes developed in this study, which exhibited a 38.274% inhibition.

This study demonstrates that the prepared phytosomes (18.877 mg GAE/mL) exhibit higher antioxidant activity than the extract alone (41.037 mg GAE/mL). Furthermore, the DPPH• scavenging activities of the extract (33.365%) and the phytosomes (38.274%) are very similar, suggesting that phytosomes can achieve the same effect at a lower concentration, thereby enabling better dose control. Despite a 54% lower phenolic load, phytosomes achieved the same DPPH activity as the free extract, underlining the assay’s usefulness for gauging encapsulation efficiency.

The encapsulation efficiency of 46% observed in this work is lower compared to the encapsulation of plant extracts in phytosomes reported by other authors (43, 74–76). However, to the best of our knowledge, this study represents the first attempt to encapsulate dragon fruit extract in a phytosomal formulation. Further research is needed to improve this efficiency, and these findings could pave the way for future research and development in this area.

A dose of 5 mg GAE/kg of dragon fruit phenolic extract exhibited weak antihyperglycemic activity, likely due to the low dosage. In contrast, phytosomes at a lower dose (2.3 mg GAE/kg) demonstrated a superior antihyperglycemic effect compared to the extract, effectively reducing blood glucose levels and showing activity comparable to the metformin group (300 mg/kg), a known anti-diabetic drug. This suggests that phytosomes could be a viable alternative to traditional medication at a significantly lower dose. The paw-edema and glucose-overload models demonstrated functional antioxidant and anti-inflammatory activities consistent with the *in vitro* DPPH ranking, thereby reinforcing its predictive value for this formulation. Nevertheless, additional assays, such as FRAP and biologically relevant reactive oxygen species (ROS) evaluations, should be considered in future research to more comprehensively assess antioxidant potential.

The AUC results (Table A7) reveal several key insights. The phytosomal formulation, with an AUC of 30,916 mg/dL·min, demonstrated the most effective glucose regulation among all groups. This suggests that phytosomes significantly enhance the bioavailability and efficacy of bioactive compounds, achieving greater antihyperglycemic effects even at a lower dose of 2.3 mg/kg. The raw extract, with an AUC of 32,945 mg/dL·min, also showed notable antihyperglycemic effects by reducing the AUC compared to the control group (33,063 mg/dL·min), though it was slightly less effective than the phytosomal formulation and comparable to metformin.

Metformin, which recorded an AUC of 31,600 mg/dL·min, outperformed the raw extract but was less effective than the phytosomes, indicating that while metformin is a reliable antihyperglycemic agent, phytosomes of extract offer a natural alternative with similar or enhanced efficacy. Finally, the control group displayed the expected natural glucose excursion over time, with an AUC of 33,063 mg/dL·min, validating the experimental design and serving as a baseline for comparison.

The dose selection in this study was informed by preliminary screening to identify an effective yet low-dose range for the phytosomal formulation (2.3 mg GAE/kg), which demonstrated superior bioactivity compared to the raw extract (5 mg GAE/kg). This enhanced efficacy, despite the lower dose, reflects improved bioavailability, possible due to encapsulation. Further pharmacokinetic profiling is needed to quantify absorption, tissue distribution, and metabolic fate of the encapsulated compounds. However, future work should include HaCaT and Caco-2 viability assays in the follow-up project that will explore chronic dosing safety.

A meta-analysis (25) in prediabetics revealed that the fasting plasma glucose reduction was significant with the use of dragon fruit, aligning with the antihyperglycaemic effect reported here. However, the effect in T2DM patients was not significant (25).

Swarup et al. (77) investigated the anti-diabetic activity of an aqueous extract of dragon fruit (*H. undatus*) in diabetic rats induced with STZ (40 mg/kg). Diabetic animals were treated orally for 5 weeks with 500 mg/kg. This study demonstrated the antihyperglycemic effects of dragon fruit extract at a dose 100 times higher than that used in our study and under different experimental conditions. Solikhah et al. (78) assessed the anti-diabetic activity of an ethanolic extract of dragon fruit peel (*H. polyrhizus*). Diabetic mice treated with the extract (300 mg/kg) for 14 days showed a reduction in glycemic levels from 366.2 ± 4.43 mg/dL to 122.6 ± 5.59 mg/dL. However, the final values were higher than those of the control group (112.6 ± 8.38 mg/dL) and the glibenclamide group (600 μg/kg; 111.8 ± 8.92 mg/dL). By contrast, our study achieves comparable efficacy at 2.3 mg GAE kg^−1^, highlighting the dose-sparing value of phytosomes. Similar dose reductions have been reported for olive-leaf (80), but this is the first example for *Hylocereus*.

To the best of the authors knowledge, this is the first study to prepare phytosomes with dragon fruit extract and evaluate their antihyperglycemic activity.

It is important to consider that the effects described in the literature were observed in diabetic rats (77, 78), whereas in this study, non-diabetic rats subjected to glucose overload were used. Future studies should include diabetic animals to better evaluate the anti-hyperglycemic effects. Additionally, exploring other encapsulation methods and optimise process parameters to narrow the size distribution and to increase the yield and concentration of phenolic compounds to enhance the biological effects here demonstrated.

In addition to functional outcomes in the paw edema model, this study evaluated levels of two key inflammatory markers, CRP and IL-6 in the animals’ blood. Compared to the negative control group, the phytosomal formulation maintained IL-6 at baseline levels (1.5 pg./mL), while the positive control group (carrageenan-induced) exhibited elevated IL-6 (2.285 ± 1.110 pg./mL). Similarly, CRP levels were slightly increased in the positive control (0.014 ± 0.015 mg/dL) but remained low in the phytosome group (0.011 ± 0.002 mg/dL), comparable to the untreated control. These findings suggest that the phytosome-encapsulated extract may exert anti-inflammatory effects by mitigating IL-6-driven responses, which are central to acute inflammation. These cytokine outcomes align with the observed reduction in paw edema and support a mechanistic link between phytosomal delivery and modulation of early inflammatory signaling pathways. CRP and IL-6 profiles provide the first evidence that the formulation modulates early inflammatory mediators. While broader profiling (e.g., TNF-*α*, IL-1β) and molecular pathway analysis (e.g., NF-κB, MAPK) were beyond the scope of this study, the current data provide a basis for hypothesizing involvement of key cytokine networks, particularly those involving IL-6. Given that key constituents (cyanidin-3-O-rutinoside, betanin, ferulic acid) are recognised NF-κB inhibitors (81–83), it is plausible that phytosomal delivery attenuates the NF-κB/IL-6 axis; this aspect needs direct pathway studies in future work. The observed reduction in carrageenan-induced paw edema suggests that the phytosomal formulation may act through known anti-inflammatory pathways involving oxidative stress modulation and cytokine suppression.

Nur et al. (84) demonstrated that aqueous extracts of dragon fruit *H. polyrhizus* (100 mg/kg) possess anti-inflammatory activity, reducing paw edema volume induced by carrageenan. Their study showed that 100 mg/kg of extract resulted in edema of 28.65, 21.35, and 23.22% at 1, 3, and 5 h, respectively, which were lower compared to the control group treated with indomethacin (20 mg/kg; 68.26, 85.28, and 98.78%, respectively). The present results surpass those of Nur et al. (84), despite using a much lower dose (5 mg/kg), overcoming the toxicity issue related to higher doses use. Moreover, the encapsulation of dragon fruit extract in phytosomes proved to be advantageous for achieving the desired anti-inflammatory effect, even with a lower dose. Compared to the results of Nur et al. (84), phytosomes at a dose of 2.3 mg GAE/kg showed similar anti-inflammatory effects than the 100 mg GAE/kg used by Nur et al (84). Telange et al. (85) developed a phytosomal formulation with quercetin, a flavonoid found in dragon fruit. They evaluated its anti-inflammatory activity using the carrageenan-induced paw edema model. The quercetin phytosomes demonstrated an anti-inflammatory effect. However, compared to the present findings, quercetin phytosomes showed a greater increase in paw volume, indicating that dragon fruit extract may have a better anti-inflammatory effect.

Dragon fruit phytosomes reduced edema more effectively than the extract alone, suggesting that phytosomes improve the bioavailability and anti-inflammatory effect of phenolic compounds. Previous studies have linked key phytoconstituents identified in the present extract, such as cyanidin-3-O-rutinoside, betanin, and ferulic acid, to the downregulation of NF-κB signaling and pro-inflammatory cytokines (81–83).

Activation of the Nrf2/ARE pathway has been observed, with recent studies on dragon fruit demonstrating effective ROS quenching by phenolic compounds such as gallic acid, ferulic acid moieties, and betanin, which act by donating hydrogen atoms or electrons to neutralise ROS (10). These findings align with the antioxidant effects noted in the DPPH assay conducted in this study. Phenolic acids have also been shown to upregulate key antioxidant enzymes, including SOD, CAT, and GPx, a phenomenon further supported by the observed *in vivo* enhancement of antioxidant enzyme expression following pitaya pulp administration (10). NF-κB inhibition related to betanin, cyanidin-3-rutinoside, and ferulic acid inhibit IκB-*α* phosphorylation and reduce TNF-α and IL-6 levels (86), which reflects in a 32% reduction in paw edema and decreased plasma IL-6 concentrations in treated rats. α-Amylase / α-Glucosidase competitive inhibition related to rutin and ferulic acid have been found to delay starch digestion (87), consistent with a 29% reduction in glycaemic peak observed in a sugar-overload model. The use of phytosome-based delivery systems, that incorporate phosphatidylcholine complexes, enhances membrane permeation and lymphatic transport of phenolics. This results in a 2.4-fold decrease in plasma AUC and more pronounced *in vivo* effects compared to the administration of free extracts. In summary, the synergistic interaction between direct redox activity, enzymatic modulation, and improved bioavailability accounts for the superior antioxidant, anti-inflammatory, and antihyperglycaemic outcomes observed in these studies.

These findings support a potential mechanistic basis that justifies further investigation in future studies focusing on molecular targets. Plasma or tissue levels of phenolic compounds like betalains and ferulic acid metabolites should also be quantified.

Therefore, encapsulating dragon fruit extract in phytosomes appears to enhance the bioavailability of the bioactive compounds present, thereby increasing their biological effectiveness.

## 5 Conclusion

Water proved to be the optimal extraction solvent, resulting in a substantial increase in yield and a higher concentration of phenolic compounds in the extract. Therefore, this solvent was used to obtain an extract rich in phenolic compounds and total flavonoids. The phenolic extract of dragon fruit contained 410.4 ± 2.57 mg GAE/L total phenolics and 139.3 ± 3.01 mg CE/L total flavonoids. LC-DAD-ESI-QqQ-MS/MS confirmed citric acid, succinic acid, ferulic acid, rutin and cyanidin-3-O-rutinoside as key constituents of the *Hylocereus costaricensis* phenolic extract. Encapsulating the extract in phytosomes (46% loading; 1.3 μm average size, PDI 0.33; *ζ* − 16 mV) preserved its antioxidant capacity and, at less than half the phenolic dose, delivered metformin-like antihyperglycaemic and superior anti-inflammatory effects in vivo. These results indicate that phytosomal delivery overcomes the extract’s intrinsic bioavailability barriers, amplifying its biological activity. Thus, pitahaya-based phytosomes represent a promising low-dose, food-grade adjunct to conventional antioxidant, anti-diabetic and anti-inflammatory interventions. Future work should optimise encapsulation efficiency and confirm long-term safety and efficacy in clinical settings.

## Data Availability

The original contributions presented in the study are included in the article/Supplementary material, further inquiries can be directed to the corresponding author.
