# Supplementary material for: Phytosomal delivery enhances bioactivity of Hylocereus costaricensis phenolic extract
Source: Front Nutr. 2025 Aug 14;12:1659572. doi: 10.3389/fnut.2025.1659572 (PMC12391043; doi:10.3389/fnut.2025.1659572)
Supplement: Supplementary file 1 [file Data_Sheet_1.docx]

Appendix A

**Table A1.** Parameters of calibration curves and total phenol dosage of dragon fruit extracts in different solvents.

| Calibration curves | | | | | TPC (mg GAE/100g FF) |
| --- | --- | --- | --- | --- | --- |
| Solvents | Concentration range (mg/L) | R² | y=mx+b | |  |
|  |  |  | m | b |  |
| Water (1:2) | 10-300 | 0.9959 | 0.0022 | 0.0152 | 165.8 ± 0.895 |
| Ethanol 50% (1:1) |  | 0.9912 | 0.0020 | 0.0729 | 17.018 ± 0.448 |
| Ethanol 50% (1:2) |  |  |  |  | 60.505 ± 0.702 |

**Table A2.** Parameters of the calibration lines and total flavonoid dosage of dragon fruit extracts in different solvents.

| Calibration curves | | | | | Total Flavonoids (mg CE/100g FF) |
| --- | --- | --- | --- | --- | --- |
| Solvents | Concentration range (mg/L) | R² | y=mx+b | |  |
|  |  |  | m | b |  |
| Water (1:2) | 20-100 | 0.9950 | 0.0039 | 0.0138 | 110.1 ± 0.673 |
| Ethanol 50% (1:1) | 20-150 | 0.9911 | 0.0032 | 0.0441 | 8.503 ± 0.305 |
| Ethanol 50% (1:2) |  |  |  |  | 22.447 ± 0.500 |

**Table A3.** Parameters of the calibration curves and dosing of total phenols and flavonoids from the aqueous extract of dragon fruit for to apply in *in vitro* and *in vivo* study.

| Calibration curves | | | | | Determination |
| --- | --- | --- | --- | --- | --- |
| Method | Concentration range (mg/L) | R² | y=mx+b | |  |
|  |  |  | m | b |  |
| TPC (mg GAE/100 mL) | 10-250 | 0.9983 | 0.0027 | 0.018 | 41.037 ± 0.257 |
| Total Flavonoids (mg CE/100g FF) | 20-80 | 0.9936 | 0.0037 | 0.016 | 13.930 ± 0.301 |

**Table A4.** Antioxidant activity (DPPH) of the extract, phytosomes and positive control (quercetin).

|  | Antioxidant activity ± SD (%) |
| --- | --- |
| Extract (41.037 mg GAE/mL) | 33.365 ± 0.402 |
| Phytosomes (18.877 mg GAE/mL) | 38.274 ± 0.819 |
| Quercetin (10 mg/mL) | 78.266 ± 0.677 |

**Table A5.** Blood glucose levels at 0 minutes (before glucose administration) and at 30, 60 and 120 minutes (after glucose administration). Four groups of rats were tested: control, metformin (300 mg/kg), extract (5 mg/kg) and phytosomes (2.3 mg/kg). The results were expressed as mean ± SD.

|  | 0 minutes | 30 minutes | 60 minutes | 120 minutes |
| --- | --- | --- | --- | --- |
| Control | 78.83 ± 9.24 | 160.5 ± 6.35 | 138.0 ± 3.46 | 103.3 ± 3.50 |
| Metformin (300 mg/kg) | 88.25 ± 2.22 | 134.3 ± 2.99 | 127.0 ± 8.60 | 109.8 ± 7.23 |
| Extract  (5 mg/kg) | 93.50 ± 4.04 | 156.3 ± 14.45 | 128.7 ± 17.80 | 111.5 ± 2.52 |
| Phytosomes (2.3 mg/kg) | 91.50 ± 4.43 | 138.0 ± 4.36 | 118.2 ± 10.89 | 98.50 ± 2.26 |

**Table A6**. Area under the curve (AUC) for blood glucose levels in control and treatment groups (*Hylocereus* formulations and metformin) over 120 minutes, demonstrating the antihyperglycemic effects of each treatment.

| Group | Area Under the Curve (mg/dL·min) |
| --- | --- |
| Control | 33063 |
| Metformin (300 mg/kg) | 31600 |
| Extract (5 mg/kg) | 32945 |
| Phytosomes (2.3 mg/kg) | 30916 |

**Table A7.** Animal paw volume at 0, 3 and 6 hours. Four groups of rats were tested: negative control (without edema induction), positive control (with edema induction), extract (5 mg/kg) and phytosomes (2.3 mg/kg). The results were expressed as mean ± SD.

|  | 0 hours | 3 hours | 6 hours |
| --- | --- | --- | --- |
| Negative control | 1.10 ± 0.01 | 1.13 ± 0.07 | 1.09 ± 0.05 |
| Positive control (carrageenan) | 1.10 ± 0.02 | 1.97 ± 0.00 | 1.65 ± 0.09 |
| Extract (5 mg/kg) | 1.20 ± 0.04 | 1.65 ± 0.05 | 1.66 ± 0.09 |
| Phytosomes (2.3 mg/kg) | 1.15 ± 0.01 | 1.42 ± 0.07 | 1.35 ± 0.03 |


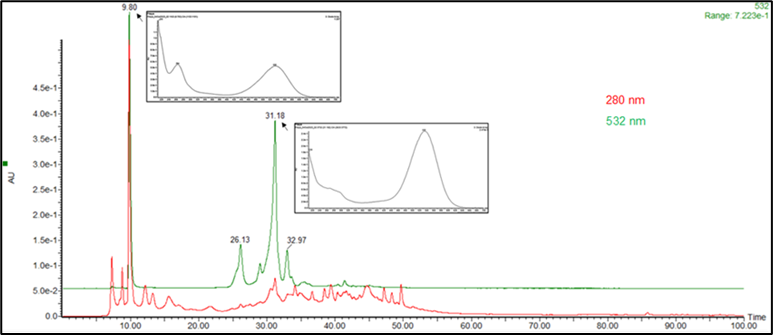


**Figure A1.** Comparison of the chromatograms at 280 and 532 nm, between 0 and 100 minutes of the dragon fruit extract. In red are the peaks corresponding to the compounds that have the highest absorption at 280 nm and in green the peaks of the compounds with the highest absorption at 532 nm. In black, spectra of absorption of compounds at 9.80 minutes and 31.18 minutes.


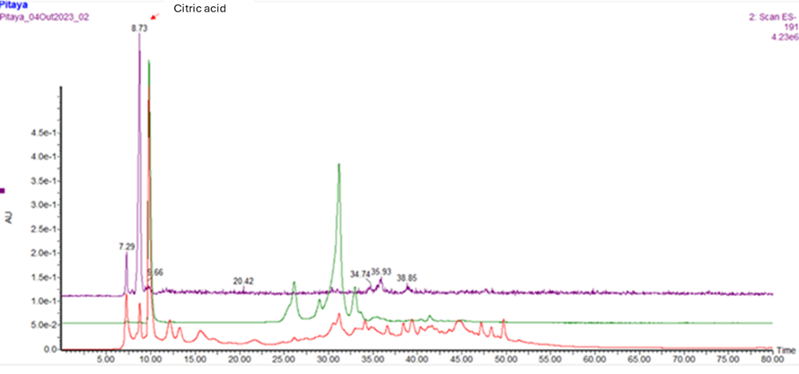


**Figure A2.** Identification of the compound with m/z 191, which corresponds to citric acid, at 8.73 minutes in dragon fruit extract. Analysis in negative mode (ESI-).


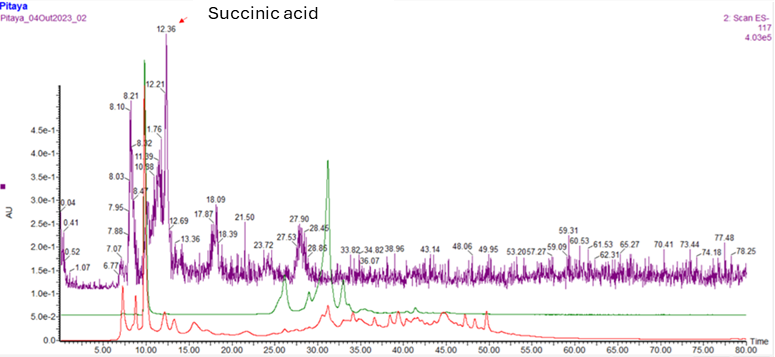


**Figure A3.** Identification of the compound with m/z 117, which corresponds to succinic acid, at 12.36 minutes in dragon fruit extract. Analysis in negative mode (ESI-).


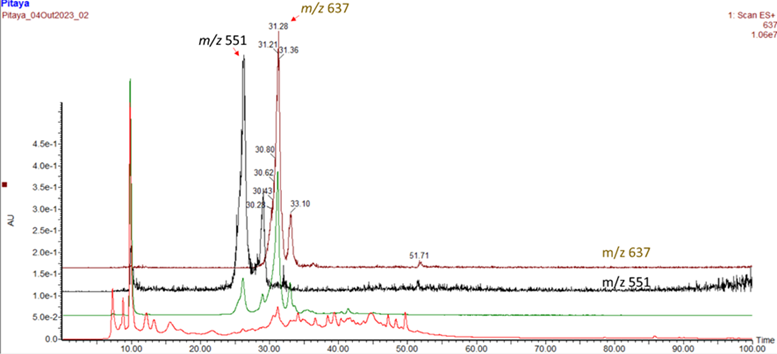


**Figure A4.** Identification of compounds with m/z 551 and m/z 637, at 26 and 31.28 minutes, respectively, in dragon fruit extract. Analysis in positive mode (ESI+).


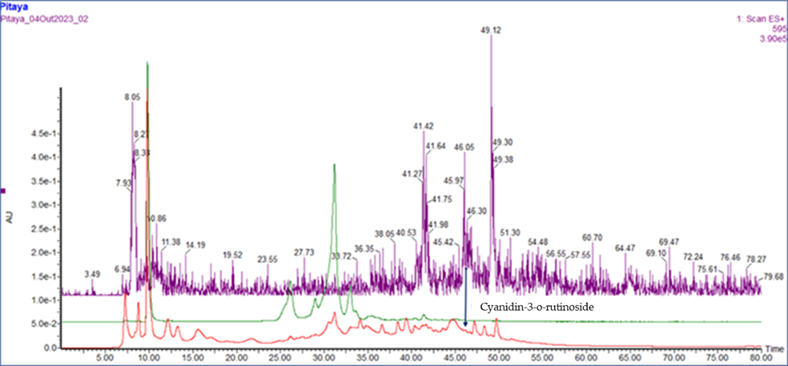


**Figure A5.** Identification of the compound with m/z 595, which corresponds to cyanidin-3-o-rutinoside, at 46.30 minutes in dragon fruit extract. Analysis in positive mode (ESI+).


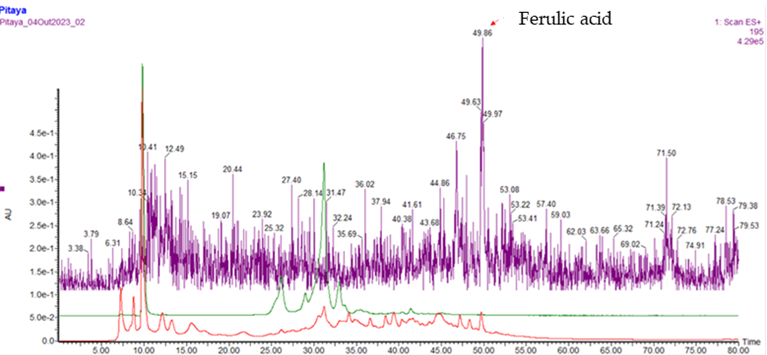


**Figure A6.** Identification of the compound with m/z 195, which corresponds to ferulic acid, at 49.86 minutes in dragon fruit extract. Analysis in positive mode (ESI+).


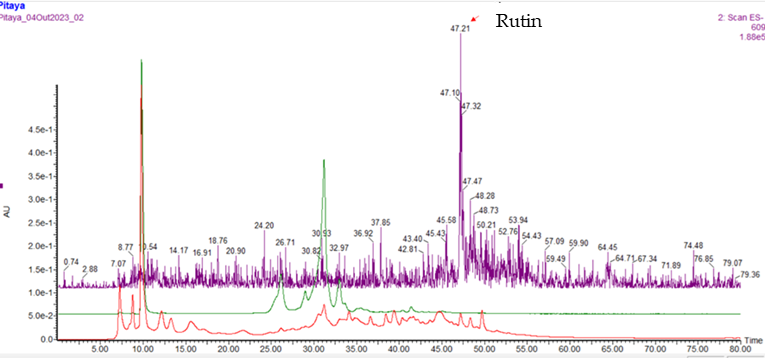


**Figure A7.** Identification of the compound with m/z 609, which corresponds to rutin, at 47.21 minutes in dragon fruit extract. Analysis in negative mode (ESI-).
